# An unsupervised learning approach to ultrasound strain elastography with spatio-temporal consistency

**DOI:** 10.1088/1361-6560/ac176a

**Published:** 2021-09-03

**Authors:** Rémi Delaunay, Yipeng Hu, Tom Vercauteren

**Affiliations:** 1 Wellcome/EPSRC Centre for Interventional and Surgical Sciences, University College London, Gower Street, London WC1E 6BT, United Kingdom; 2 School of Biomedical Engineering & Imaging Sciences, King’s College London, Strand, London WC2R 2LS, United Kingdom

**Keywords:** ultrasound, elastography, quasi-static, recurrent neural network, LSTM, unsupervised, deep learning

## Abstract

Quasi-static ultrasound elastography (USE) is an imaging modality that measures deformation (i.e. strain) of tissue in response to an applied mechanical force. In USE, the strain modulus is traditionally obtained by deriving the displacement field estimated between a pair of radio-frequency data. In this work we propose a recurrent network architecture with convolutional long-short-term memory decoder blocks to improve displacement estimation and spatio-temporal continuity between time series ultrasound frames. The network is trained in an unsupervised way, by optimising a similarity metric between the reference and compressed image. Our training loss is also composed of a regularisation term that preserves displacement continuity by directly optimising the strain smoothness, and a temporal continuity term that enforces consistency between successive strain predictions. In addition, we propose an open-access *in vivo* database for quasi-static USE, which consists of radio-frequency data sequences captured on the arm of a human volunteer. Our results from numerical simulation and *in vivo* data suggest that our recurrent neural network can account for larger deformations, as compared with two other feed-forward neural networks. In all experiments, our recurrent network outperformed the state-of-the-art for both learning-based and optimisation-based methods, in terms of elastographic signal-to-noise ratio, strain consistency, and image similarity. Finally, our open-source code provides a 3D-slicer visualisation module that can be used to process ultrasound RF frames in real-time, at a rate of up to 20 frames per second, using a standard GPU.

## 1. Introduction

### 1.1. Background

Ultrasound elastography (USE) is an imaging modality that enables the characterisation of the elastic properties of tissue (Sigrist *et al* 2017). Mapping tissue elasticity is particularly useful in diagnostic applications, where the presence of pathology can cause modifications in tissue stiffness. It includes the characterisation of lesions in different organs, such as the liver (Ferraioli *et al* 2015) or prostate (Moradi *et al* 2007), but also differentiation between benign and malignant tumours, such as those found in the thyroid (Hong *et al* 2009) and breast (Hall *et al* 2003). USE has also shown promising results in image-guided interventions, including liver resection (Kato *et al* 2008, Otesteanu *et al* 2018) and brain tumour surgery (Chakraborty *et al* 2012).

This work focuses on quasi-static, free-hand palpation elastography, where a time-varying axial compression is applied to the target tissue, using a handheld ultrasound probe (Ophir *et al* 1991, Varghese 2009). In quasi-static elastography, the mechanical behaviour of a tissue is determined by mapping the relative deformation (i.e. strain) induced by manual compression (i.e. stress). The strain is generally obtained by deriving the displacement between a pair of ultrasound radio-frequency data before and after applying a quasi-static deformation on the tissue. Even though quasi-static elastography does not provide a quantitative measure of tissue elasticity (e.g. the Young’s modulus), the strain information can be a useful adjunct to conventional ultrasound, because the echogenic properties of tissues and their stiffness are not necessarily correlated. In addition, there are no specific hardware requirements for generating the mechanical excitation in USE, unlike dynamic USE methods, such as shear wave elastography or acoustic radiation force imaging (Sigrist *et al* 2017). Therefore, USE can be used with most clinical ultrasound scanners, making it highly portable and relatively cost effective.

### 1.2. Related work

Strain information is obtained by computing the spatial gradient of the displacement field, making speckle tracking a key processing step in quasi-static elastography. Various methods of displacement estimation have been proposed over the years. Historically, it has been performed by maximising a correlation function between local frame windows, either in the time or phase domain (Ophir *et al* 1996, Varghese *et al* 2000, Azar *et al* 2010, Alessandrini *et al* 2014). Although windows-based methods have shown good performance in displacement estimation, working with local windows prevents the accurate prediction of large deformation and decreases robustness to global decorrelation, i.e. the change of speckle appearance due to out-of-plane motion. A different strategy, which can be referred to as optimisation-based methods, involves minimising a cost function that combines image similarity and displacement regularity (Pellot-Barakat *et al* 2004, Kuzmin *et al* 2015, Hashemi and Rivaz 2017). These methods assume the displacement throughout the tissue to be smooth and, therefore, justify the use of a regularisation parameter that penalises the correlation function to prevent displacement discontinuity. However, this type of approach can be computationally expensive and is not suitable for real-time application.

Recent methods have adopted the use of deep neural networks for USE, and have demonstrated high accuracy and robustness in displacement estimation. Most of these methods share the same general training strategy, which minimises a supervised loss function between the network’s displacement estimates and their respective ground truth labels, generated from numerical ultrasound phantoms via finite element methods (FEM) (Kibria and Rivaz 2018, Wu *et al* 2018, Gao *et al* 2019, Peng *et al* 2020, Tehrani and Rivaz 2020). This learning strategy prevents the model from training on real-world ultrasound data because ground truth displacement fields are not possible to obtain when the magnitude of applied stress is unknown. Moreover, learning from real-world ultrasound data can improve the model’s generalisation ability because this data often exhibits complex speckle patterns and echogenic features, which can be quite challenging to replicate in ultrasound simulation.

Alternative approaches adopted networks trained with unsupervised algorithms, which allow a model to be fine-tuned directly on any given radio-frequency ultrasound data and dispense the need to use ground truth labels. Learning displacement estimation in an unsupervised way has been successfully applied to medical image registration techniques (de Vos *et al* 2017, Balakrishnan *et al* 2019). The basic principle consists in using a loss function which captures the image similarity between the reference and the warped moving image, and the displacement continuity; rather than computing the difference between the output and some ground truth. In the case of quasi-static elastography, a semi-supervised method was proposed (Tehrani *et al* 2020) that fine-tuned a pre-trained optical flow network (LiteFlowNet) on ultrasound phantom data, using an unsupervised training scheme. In previous work, we also introduced an end-to-end unsupervised approach, where a model was directly trained with *in vivo* data by using ultrasound images of the arm of human volunteers (Delaunay *et al* 2020).

Another research direction in quasi-static elastography aims to find the most suitable pair of images to be used for strain estimation. Quasi-static elastography only requires two image frames to estimate the strain modulus, but the resulting information is not always relevant. A non-uniform or small axial compression occurring between an image pair can greatly affect the signal-to-noise ratio (SNRe) and result in a strain map that does not effectively characterise the tissue stiffness. A common solution to this problem is to compute the strain between all image pairs and associate each resulting strain with a confidence score based on image similarity (Jiang *et al* 2006, Treece *et al* 2011) and/or tracking information (Foroughi *et al* 2013). In Zayed and Rivaz (2020), the frame selection is performed before displacement estimation by using a classifier that gives a binary decision on the suitability of the image pair for strain computation.

Finding the best image pair also means searching for the optimal interframe interval, i.e. the time interval between successive ultrasound frames, which greatly impacts the displacement estimation. A high interframe interval exacerbates decorrelation noise due to physiologic motion, such as blood flow and muscle movement, which can greatly affect the performance of displacement estimation methods and reduce the quality of the resulting strain map. Therefore, this limits the range of possible image pairs in the temporal dimension for frame-pairing methods (Chandrasekhar *et al* 2006). Furthermore, commercial scanners can acquire images at a high-frame-rate and frame-pairing strategies discard a large proportion of the available data. The strain image quality can also be improved by accumulating successive displacement fields (Varghese and Ophir 1996, Lubinski *et al* 1999) or by normalising strain images with an estimate of the applied stress (Lindop *et al* 2008).

### 1.3. Contributions

In this paper, we present an end-to-end unsupervised learning-based method for quasi-static elastography that allows a neural network to be trained directly on readily-available clinical data. Our training procedure does not use ground truth labels and allows a model to be fine-tuned only using RF ultrasound data as input. The network weights are optimised by minimising a dissimilarity function between the pre-compression and warped compressed images.

**Figure 1.**
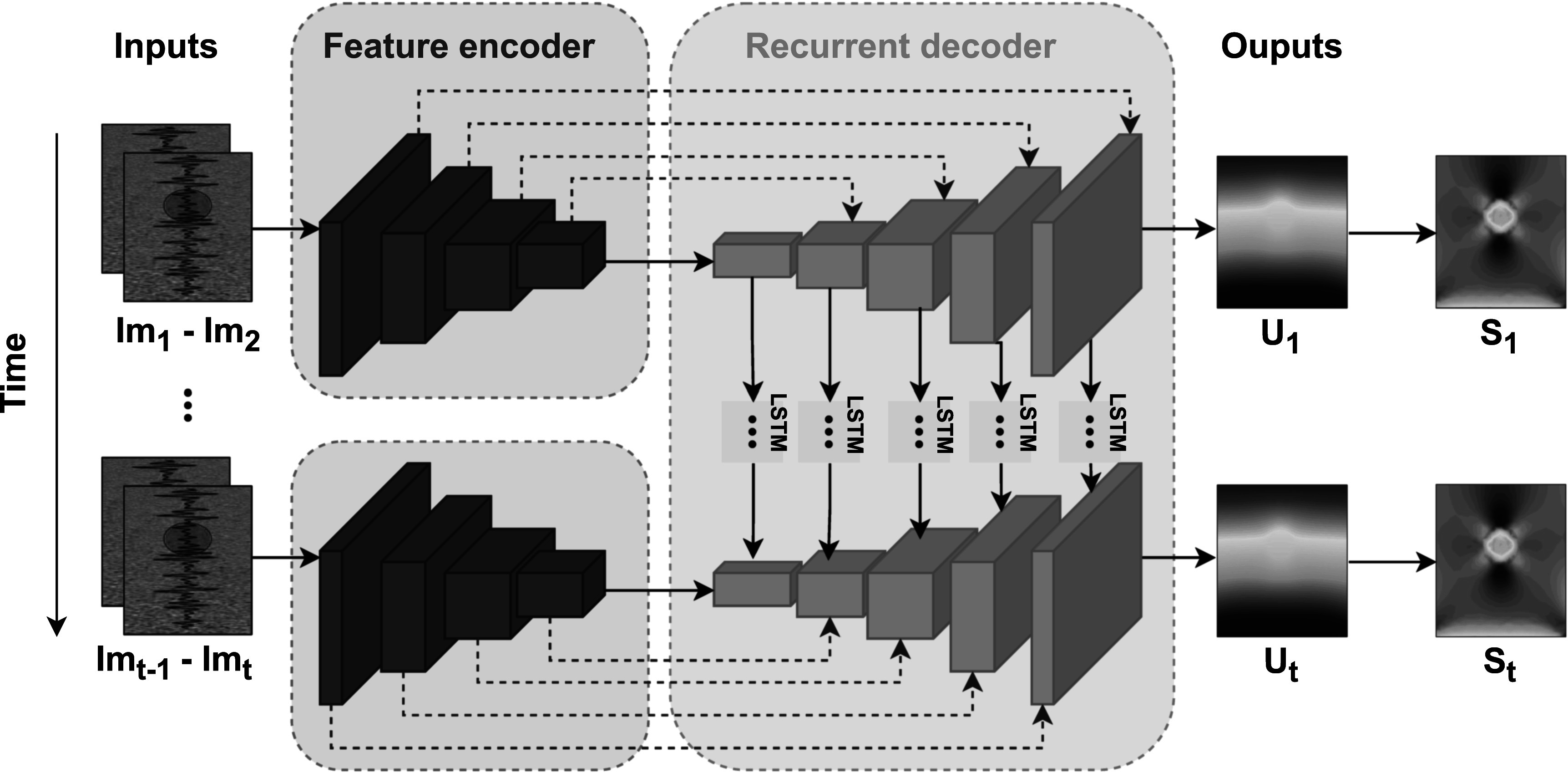
Overview of the proposed recurrent model for ultrasound strain elastography (ReUSENet). At each time step *t*, the network takes as inputs a pair of radio-frequency data frames (${{\mathrm{Im}}}_{t-1},{{\mathrm{Im}}}_{t}$) and outputs the dense displacement field *U*_*t*_ between the inputs images. The displacement field is then spatially derived to the strain field, by using the least-squares strain estimator (LSQSE).

In addition, we propose a novel network architecture based on convolutional long-short-term memory units (convLSTM) (Xingjian *et al* 2015) to improve displacement estimation accuracy for image pairs that are temporally distant, by making use of all the intermediate frames. The use of intermediate ultrasound frames improved the displacement estimation of our recurrent network for large range deformations, as well as the consistency between consecutive strain predictions. We called our method ReUSENet, which stands for recurrent ultrasound strain elastography network. At inference, ReUSENet takes a temporal sequence of RF ultrasound data as input and predicts the displacement and strain maps of consecutive image pairs by making use of the memory state of the convLSTM units captured from previous predictions. An overview of ReUSENet is presented in figure 1.

We compare the performance of ReUSENet with a standard feed-forward neural network architecture, named here unsupervised strain elastography network (USENet). We validated our two models on numerical simulation and *in vivo* data, and compared our results to state-of-the-art deep learning-based and optimisation-based algorithms (Hashemi and Rivaz 2017, Tehrani and Rivaz 2020). Both networks can be run in real-time at a speed of about 20 frames per second with a standard 12 GB GPU. The contribution of our paper can be summarised as follow:

• We propose an end-to-end unsupervised method, which allows models to be trained directly on *in vivo* data.
• We propose the first recurrent neural network applied to quasi-static elastography to improve both displacement estimation accuracy and strain image quality between temporally distant ultrasound frames.
• We provide an open-access, publicly available *in vivo* database which consists in 17 271 RF data of blood vessels from the arm of a human volunteer.^3^ 
^3^
Open-access database available on https://synapse.org/InVivoDataForUSE

• We provide an open-source 3D-slicer extension called DeepUSE, which has been designed to perform real-time inference with the networks introduced in the paper, for both USENet and ReUSENet.^4^ 
^4^
Slicer DeepUSE module available at https://github.com/RemiDelaunay/SlicerDeepUSE

## 2. Methods

### 2.1. Network architectures

#### 2.1.1. USENet

The architecture of our feed-forward network is based on the U-Net (Ronneberger *et al* 2015), which consists of an encoder–decoder convolutional neural network with skip connections. The use of this type of architecture has been demonstrated successfully for optical flow estimation (Dosovitskiy *et al* 2015), but also for many medical image registration tasks (Hu *et al* 2018 Balakrishnan *et al* 2019).

The encoder part is composed of four down-sampling ResNet blocks (He *et al* 2016), which capture the hierarchical features necessary to establish correspondence between the pair of images. Each block corresponds to a residual unit composed of two sequential convolutional layers with a batch normalisation layer and leaky rectified linear unit. Max pooling is performed after each ResNet block to reduce the dimension of the extracted features.

Symmetrically, the decoder part is composed of four up-sampling blocks that consists of an additive up-sampling layer summed over a transpose convolutional layer. Finally, each up-sampling block outputs a displacement field that is convolved and resized to the input size, then summed to output the predicted displacement field.

#### 2.1.2. ReUSENet

**Figure 2.**
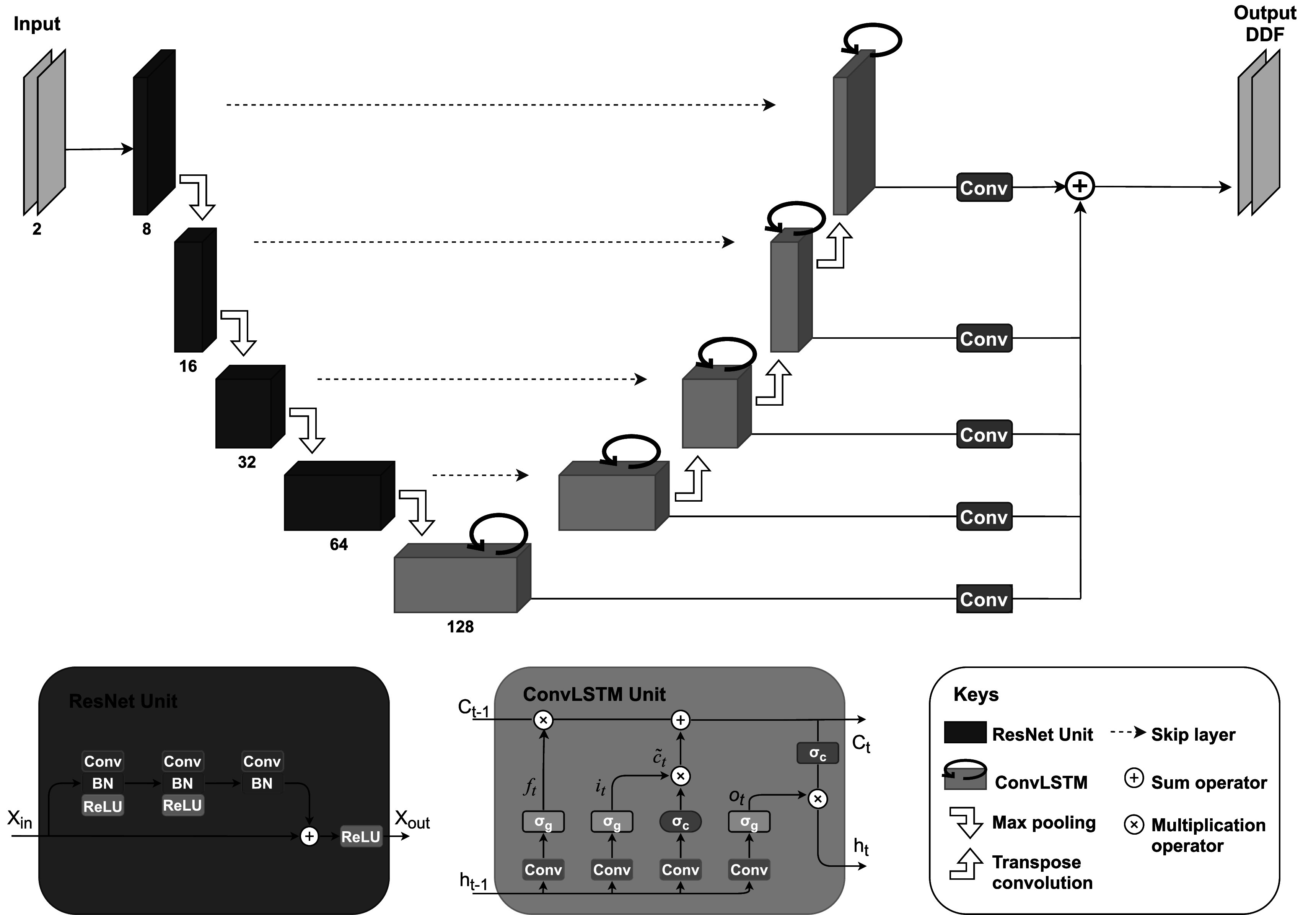
Network architecture of ReUSENet. Each blue rectangle constitutes the encoder part of the network and corresponds to a ResNet unit (illustrated bellow) with different channel size printed underneath. The decoder is composed of successive convLSTM units which are represented in orange and described in details at the bottom of the figure. The output of each convLSTM unit is then convolved and resized to the input size before being summed to output the displacement field.

The network architecture of ReUSENet is presented in figure 2. The encoder part of the recurrent network is the same as USENet. In the decoder part, the up-sampling blocks from USENet are replaced by convLSTM units (Xingjian *et al* 2015). LSTMs are a type of neural network that have been designed to learn long-term dependencies and process temporal sequences of data (Hochreiter and Schmidhuber 1997). A standard LSTM unit is composed of a memory cell *c*_*t*_, also known as the internal state, and three ‘gates’ regulating the flow of information, i.e the input gate *i*_*t*_, output gate *o*_*t*_ and forget gate *f*_*t*_. Intuitively, the memory cell keeps track of the dependencies between the inputs of the temporal sequence, the input gate controls the incoming input flow, the forget gate controls the amount of information to keep in the cell and the output gate controls the amount of information to use for the output. The output of an LSTM is called the hidden state and is noted *h*_*t*_.

A convLSTM cell differs from a standard LSTM unit by taking multi-dimensional data as input, such as videos. This is done by replacing the fully-connected layer of each gate by a convolution operation to capture the image spatial features. The updated equations can be written as follow:

\begin{eqnarray*}\left\{\begin{array}{l}{i}_{t}={\sigma }_{g}({x}_{t}* {W}_{{xi}}+{h}_{t-1}* {W}_{{hi}}+{b}_{i})\\ {f}_{t}={\sigma }_{g}({x}_{t}* {W}_{{xf}}+{h}_{t-1}* {W}_{{hf}}+{b}_{f})\\ {o}_{t}={\sigma }_{g}({x}_{t}* {W}_{{xo}}+{h}_{t-1}* {W}_{{ho}}+{b}_{o})\\ {\tilde{c}}_{t}={\sigma }_{c}({x}_{t}* {W}_{{xc}}+{h}_{t-1}* {W}_{{hc}}+{b}_{c})\\ {c}_{t}={c}_{t-1}\odot {f}_{t}+{i}_{t}\odot {\tilde{c}}_{t}\\ {h}_{t}={o}_{t}\odot {\sigma }_{c}({c}_{t})\end{array}\right.,\end{eqnarray*}

where * and ⊙ correspond respectively to a convolution operation and element-wise product. *σ*_*g*_ and *σ*_*c*_ are the logistic sigmoid and hyperbolic tangent functions. ${\tilde{c}}_{t}$ denotes the cell input activation vector. W_**_ and b_*_ correspond to the weight matrices and bias vector parameters, which are learned during training.

Inspired by Salvador *et al*, the encoded features along with the previous hidden state are fed to a convLSTM layer, which is then followed by four up-sampling convLSTM blocks (Salvador *et al* 2017). For a time step *t*, a convLSTM block *i* takes as input its temporal hidden state *h*_*i*,*t*−1_ as well as the previous spatial hidden state *h*_*i*−1,*t*_, which is up-sampled by a bilinear additive layer (Wojna *et al* 2019) and a transpose-convolution layer that are then added to the output of the symmetric encoding block output via a skip layer. Finally, each convLSTM block outputs a displacement field that is convolved and resized to the input size, then summed to output the predicted displacement field.

### 2.2. Training

The encoder of both ReUSENet and USENet takes a pair of pre- and post-compression 2D RF frames as input, here named *Pre* and *Post*, and predicts a dense displacement field. The parameters of our network are estimated by minimising a weighted loss function over the training set. The loss function is composed of an image similarity term, a displacement regularisation term and a temporal consistency term which can be written as follow:

\begin{eqnarray*}{L}_{{total}}={L}_{{sim}}+\alpha {L}_{{reg}}+\beta {L}_{{cons}}.\end{eqnarray*}

For any given training pair, the *L*_*sim*_ term is chosen as a negative local normalised cross-correlation (LNCC) function which averages the NCC score between sliding windows sampled from the pre-compression image and the post-compression image resampled with the predicted displacement field *u*. The NCC between two local image windows, *W*_1_ and *W*_2_, with *i*, *j* pixel components can be written as:

\begin{eqnarray*}{LNCC}=\displaystyle \frac{1}{N}\sum _{i,j}\displaystyle \frac{\left[{W}_{1}(i,j)-{\mu }_{{W}_{1}}\right]\times \left[{W}_{2}(i,j)-{\mu }_{{W}_{2}}\right]}{{\sigma }_{{W}_{1}}\times {\sigma }_{{W}_{2}}},\end{eqnarray*}

where *N* is the number of pixels indexed by location (*i*, *j*) and *μ* and *σ* correspond to the mean and standard deviation of the images, respectively.

Given the LNCC, the similarity loss *L*_*sim*_ can be expressed as:

\begin{eqnarray*}{L}_{{sim}}={LNCC}({Pre},{Post}\circ T),\end{eqnarray*}

where *T* corresponds to the spatial transformation predicted by the network and applied to the post-compression image to map it in the pre-compression image space.

The regularisation term corresponds to the L1-norm of the strain spatial gradient. Given that the strain modulus is defined as the displacement gradient, the strain field gradient corresponds to the second-order derivative of the predicted displacement and can be written as follows:

\begin{eqnarray*}{L}_{{reg}}=\sum _{i,j}\ (| \ {\partial }_{x}^{2}{u}_{i,j}\ | +| \ {\partial }_{x}{\partial }_{y}{u}_{i,j}\ | +| \ {\partial }_{y}^{2}{u}_{i,j}\ | +| \ {\partial }_{y}{\partial }_{x}{u}_{i,j}\ | ),\end{eqnarray*}

where *u* is the predicted axial displacement field and ${\partial }_{x}^{2}u$, ∂_*x*_∂_*y*_ *u*, ${\partial }_{y}^{2}u$ and ∂_*y*_∂_*x*_ *u* are the second-order partial derivatives of *u*.

After displacement estimation, the axial strain map is computed directly during training. In USE, the strain estimates are obtained by computing the displacement field gradient. However, direct differentiation of the displacement field is rarely used because gradient operations generate a significant amount of noise in the resulting strain map. We used the least-squares strain estimator (LSQSE) to improve the elastogram SNRe (Kallel and Ophir 1997).

Similar to our similarity loss, the strain consistency term computes the negative LNCC score between successive strain fields computed from a temporal sequence. It compares the current strain field *S*_*t*_ with the previously computed strain field *S*_*t*−1_ mapped into the coordinate system of the current field. Since the strain image is formed at the physical grid of the post-compression image, the same spatial transformation is used to perform the strain image mapping

\begin{eqnarray*}{L}_{{cons}}={LNCC}({S}_{t-1},{S}_{t}\circ T).\end{eqnarray*}

Our consistency term is inspired by previous work, where it has been used as a metric to estimate the consistency between consecutive strain frames (Jiang *et al* 2006). Jiang *et al* motivated the use of this consistency metric by assuming that noise in the strain image is uncorrelated with its underlying signal. Therefore, they suggest that a high correlation score between consecutive motion-compensated strain images indicates a relatively low noise level and consequently an improved image quality. This term is used only for the recurrent network, which deals with consecutive image pairs. Therefore, *β* is set to zero when training the USENet.

### 2.3. Implementation details

The presented method was implemented in PyTorch^5^ 
^5^
Fine-tuning code and pre-trained models are available at https://github.com/RemiDelaunay/DeepUSE
 and the following experiments were performed using a 12 GB NVIDIA GTX-1080ti GPU. The network’s weights for both USENet and ReUSENet were fine-tuned independently for the numerical and *in vivo* databases. During training, the learning rate was initialised to 1e-3 and was reduced by a factor of 0.8 when the validation loss stagnated for 10 epochs. The training was stopped when the difference between the new and previous learning rate was smaller than 1e-8. The regularisation loss weight was empirically set to *α* = 5, while the consistency weight was set to *β* = 0.2 for ReUSENet. In inference, the strain map prediction rate reached a total of 20 images per second.

## 3. Experiments

### 3.1. Experiments on numerical phantoms

We first performed a quantitative comparison on numerical simulation of both USENet and ReUSENet together with two state-of-the-art elastography methods, namely RF modified pyramid, warping and cost volume network (RFMPWC-Net) (Tehrani and Rivaz 2020) and global ultrasound elastography (GLUE) (Hashemi and Rivaz 2017). GLUE is an optimisation-based approach that relies on a regularised cost function to perform displacement estimation. We used the public Matlab implementation of GLUE to compute our results. RFMPWC-Net corresponds to a modified version of the well-known optical flow network PWC-Net (Sun *et al* 2018). We used the publicly available demo code and trained weights of the RFMPWC-Net for comparison. The network’s weights have been fine-tuned in a supervised way using an ultrasound simulation database the authors made publicly available, ‘ultrasound simulation database for deep learning’ (Tehrani and Rivaz 2020)^6^ 
^6^
The ultrasound simulation database, GLUE and RFMPWC-Net are available at https://users.encs.concordia.ca/~impact/
.

For reproducibility, we used the same ultrasound simulation database to train both USENet and ReUSENet. The database consists of 24 different phantoms with 10 different average strain values (from 0.5% to 4.5% ) and 10 different simulations with different scatterer positions, which results in a total of 2400 simulated images. The displacements were obtained by FEM using the ABAQUS software. The ultrasound images were simulated with a centre frequency of 5 MHz by using the publicly available field-II Matlab toolbox. Each digital phantom contains one or two inclusions with random positions and Young’s modulus (from 40 to 60 kPa). The first 20 numerical phantoms were used for training, whereas the last four were used for testing. To mimic a temporal stack of ultrasound imaging data, the testing dataset consisted in sequences composed of 10 ultrasound images with an increasing axial compression, i.e with average strain value ranging from 0.5% to 4.5%. All the numerical phantoms contained 10 different ultrasound simulations with different scatterer positions except for the last one, which only had 6. Therefore, the testing dataset consisted of 36 sequences of 10 images.

During training, the entire sequence was fed to ReUSENet at each iteration, while the interframe interval was randomly assigned for USENet. Strain image quality was assessed in terms of normalised root mean squared error (NRMSE) and elastographic SNRe (Islam *et al* 2018). Both similarity and consistency scores are also displayed in figure 4. NRMSE corresponds to:

\begin{eqnarray*}{NRMSE}=\sqrt{\displaystyle \frac{\sum _{i}^{N}{\left({{Predicted}}_{i}-{{Label}}_{i}\right)}^{2}}{N}}\times \displaystyle \frac{100\times N}{\sum _{i}^{N}({{Label}}_{i})},\end{eqnarray*}

where *Predicted* and *Label* are the axial displacement from the evaluated method and ground truth label, respectively. In addition, the SNRe can be written as:

\begin{eqnarray*}{SNRe}=\displaystyle \frac{\mu }{\sigma },\end{eqnarray*}

where *μ* and *σ* are the mean and standard deviation of the strain image.

**Figure 3.**
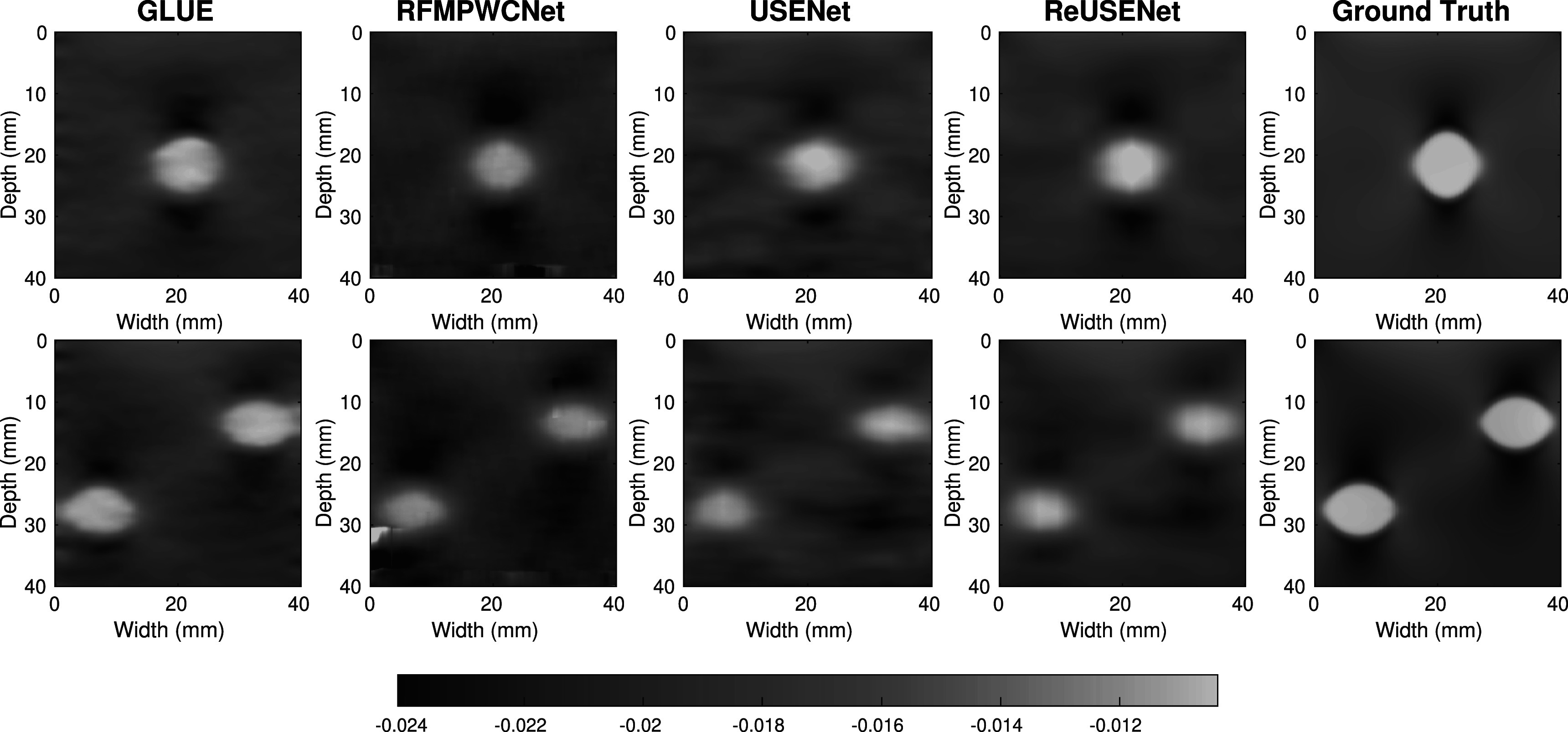
Strain images of two numerical phantoms with one inclusion (top row) and two inclusions (bottom row).

**Figure 4.**
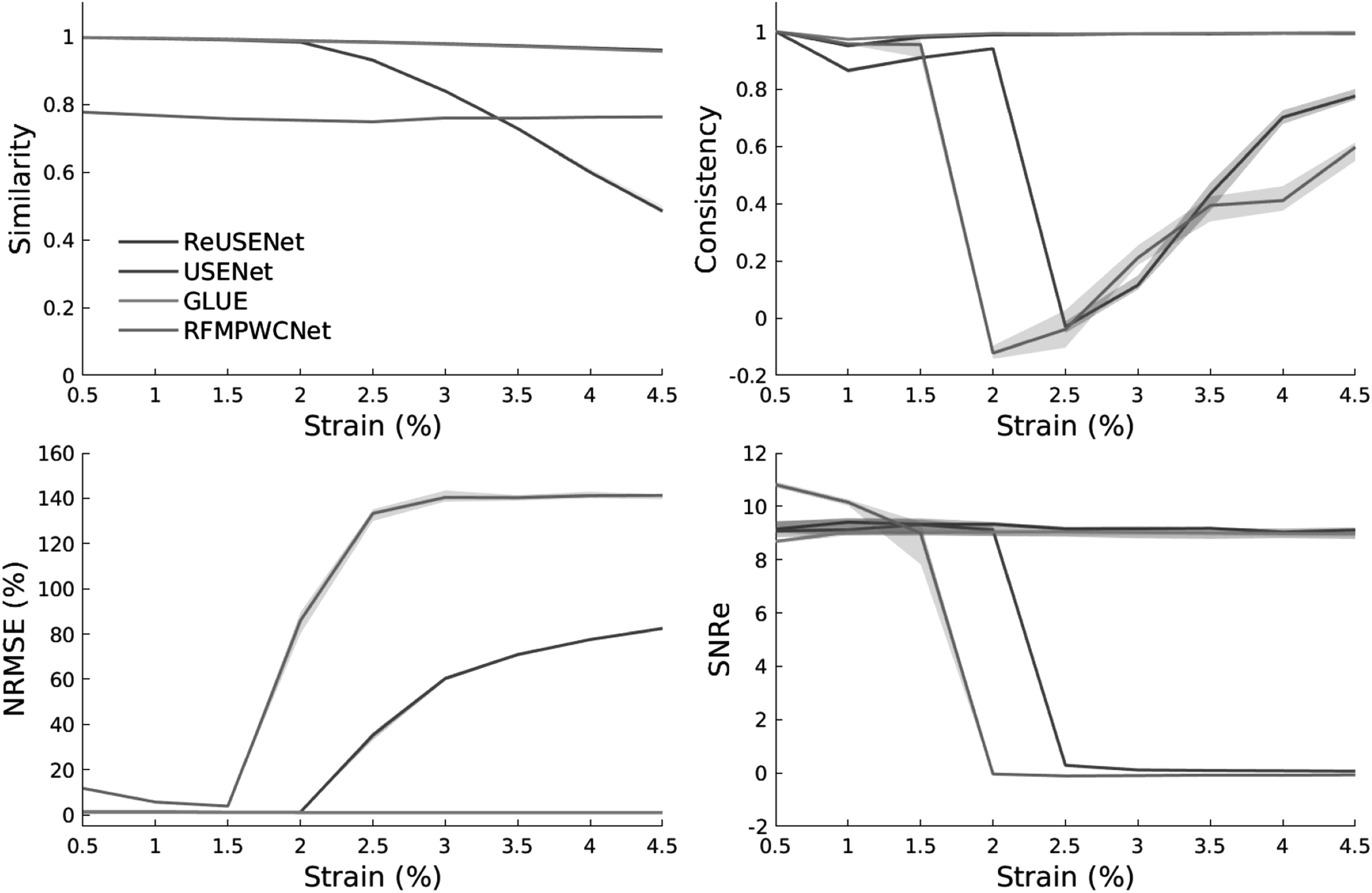
Similarity, consistency, SNRe and NRMSE scores with 25th percentiles of the compared methods for the testing simulation dataset (*N* = 36) according to strain (in %).

**Figure 5.**
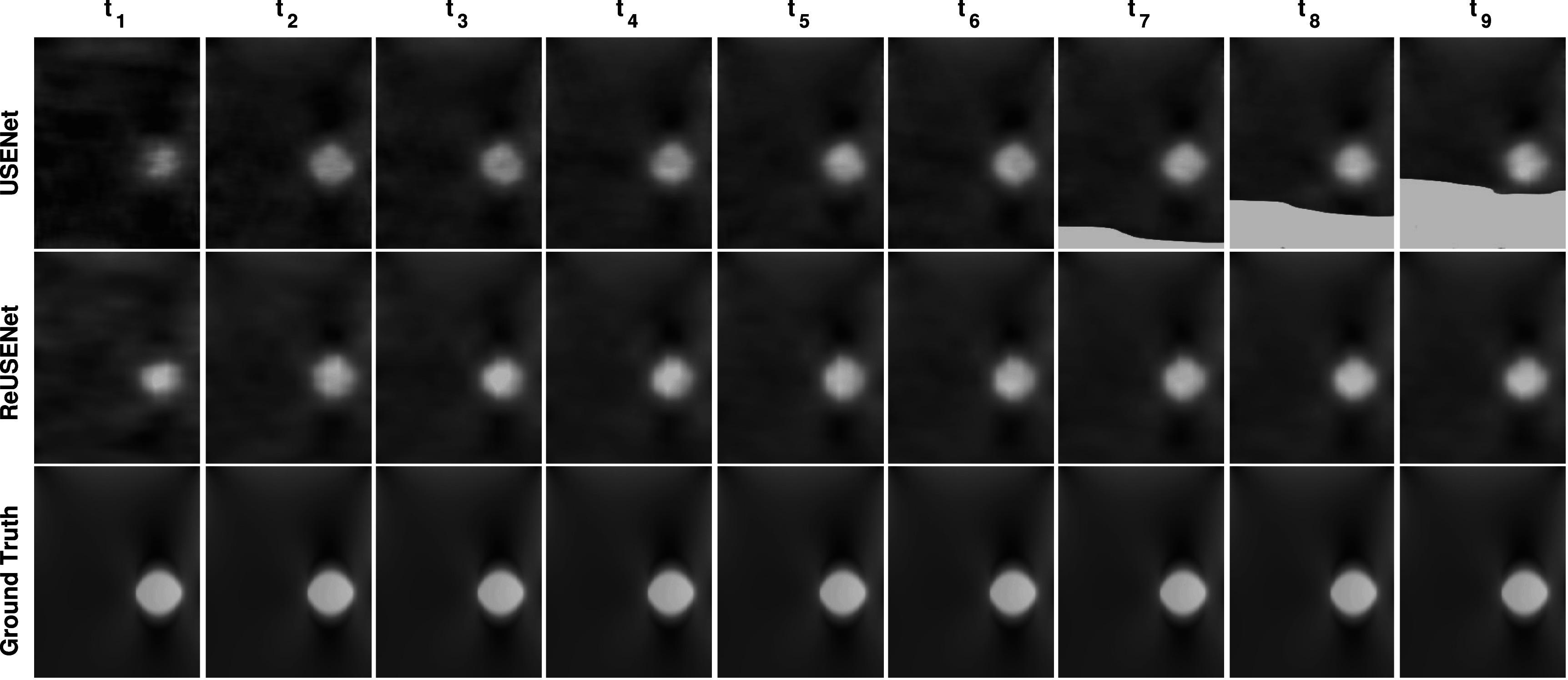
Strain images of a numerical phantom temporal sequence with increasing deformation (from 0.5% to 4.5% of strain).

An example strain image of a simulated phantom with 1.5% of average strain computed by the compared methods is shown in figure 3. Figure 4 shows the different metric score values plotted against the relative deformation (in % of strain) for the different methods. The shaded plots correspond to the average scores (in bold) with 25th percentiles (shaded). Both feed-forward neural networks (RFMPWCNet and USENet) failed to compute an accurate displacement field for large compression. The NRMSE, SNRe, similarity and consistency scores drops significantly after an average axial strain of 3.5% for USENet and 1.5% for RFMPWCNet. In contrast, both ReUSENet and GLUE provide consistent and accurate results for all compression levels. Although GLUE has the lowest variance, average scores for ReUSENet (similarity = 0.98 ± 0.001, consistency = 0.98 ± 0.003, NRMSE = 1.02 ± 0.05, SNRe = 9.19 ± 1.10) are similar or slightly better than GLUE (similarity = 0.98 ± 0.001, consistency = 0.98 ± 0.001, NRMSE = 1.10 ± 0.035, SNRe = 8.87 ± 1.046). Finally, an example of a temporal axial strain estimation sequence (from 0.5% to 4.5% strain) computed with USENet and ReUSENet is compared to ground truth simulations in figure 5 to illustrate the degradation of performance of USENet with increasing compression.

### 3.2. Experiments on *in vivo* human data

The dataset we used in the following experiment consisted of image sequences acquired from the arm of a human volunteer. Data collection was approved by the King’s College London Research Ethics Management Application System, ref HR-18/19-8881. The data was acquired by imaging the volunteer’s arm while slowly applying an axial compression with the handheld ultrasound probe. We acquired raw channel ultrasound data from a Cicada 128PX system equipped with a 7.5 MHz linear probe from Cephasonics (Cephasonics Inc., USA). The images were generated using the delay-and-sum beamformer from SUPRA (Göbl *et al* 2018).

The *in vivo* dataset included 310 sequences of variable length, i.e. from 19 to 127 images, for a total of 17 271 images. The large variance in the image sequence size can be explained by the frame-rate, which varied from 10 to 20 frame-per second, and the time it took to perform the axial compression. Not all sequences exhibit a specific targeted region with a notable difference of stiffness. For instance, a sequence can only show longitudinal muscle fibres of the forearm being compressed. We also decided to keep sequences with a large amount of lateral displacement or decorrelation noise for training our networks. However, each sequence selected for testing targeted at least one blood vessel. The *in vivo* dataset generally exhibits higher displacement and decorrelation noise between each frame as compared with the simulated dataset. Therefore, the temporal sequences used as input for ReUSENet during training and inference corresponded to 6 successive frames. A total of 20 sequences of 6 images were used for testing, sampled from 13 different acquisition sequences.

The quality of the strain estimates were assessed in terms of consistency, similarity and SNRe. Since ground truth labels were not available, we further investigated the registration accuracy by computing a target registration error (TRE) on each cases from the *in vivo* dataset. We manually identified 8 pairs of different corresponding landmarks between the first and last ultrasound frames in each temporal sequence. The TRE was then computed before and after resampling the last ultrasound frames of the sequence into the first one, by using the output displacement field predicted by USENet, GLUE and ReUSENet. The mean TRE for each cases, measured in pixels, is summarised in table 1. In order to avoid unfair comparison, RFMPWCNet was not included in this performance comparison because the network’s weights were not fine-tuned on the *in vivo* dataset, unlike USENet and ReUSENet.

**Figure 6.**
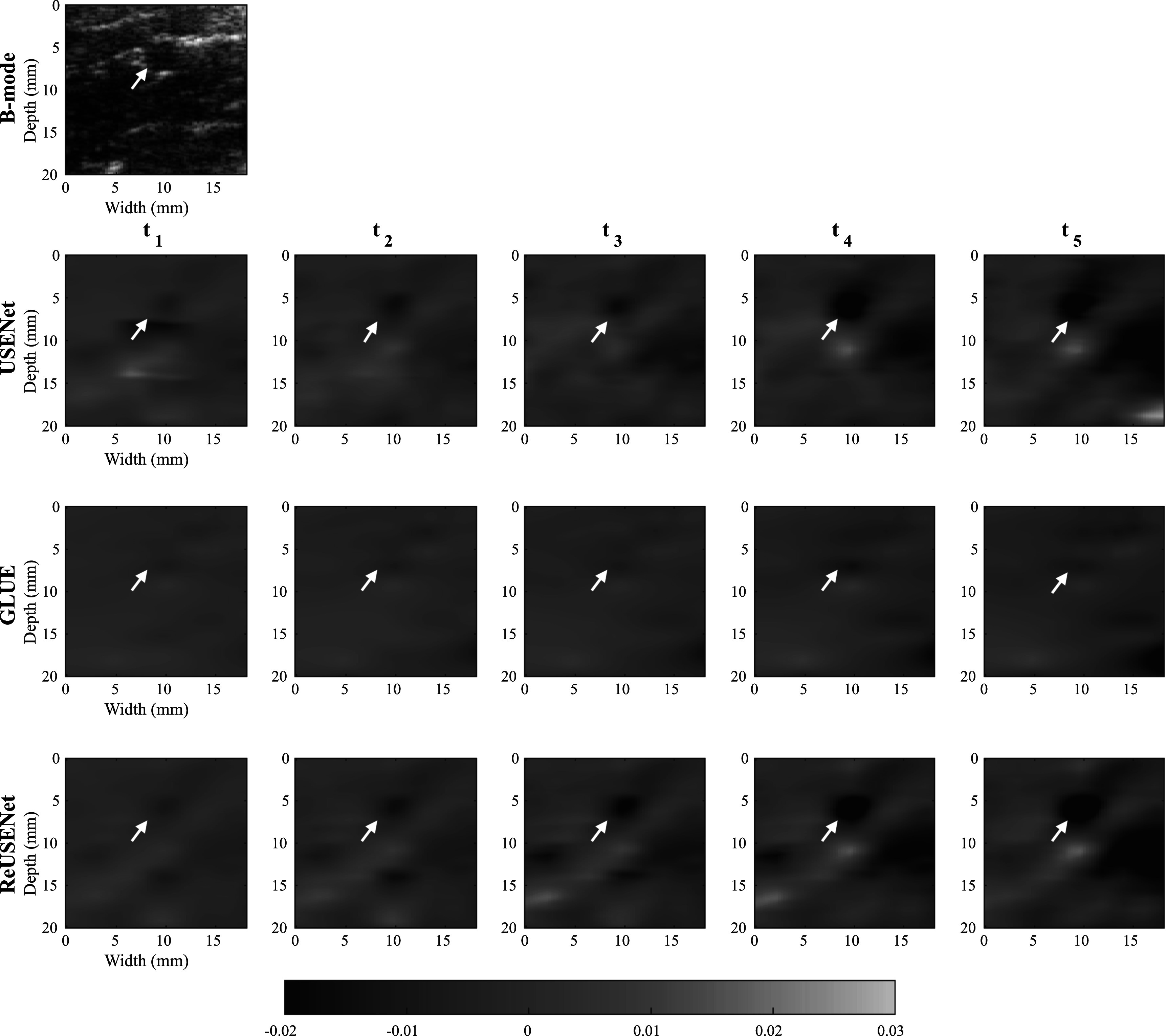
Temporal strain image sequence from the testing *in vivo* dataset.

**Figure 7.**
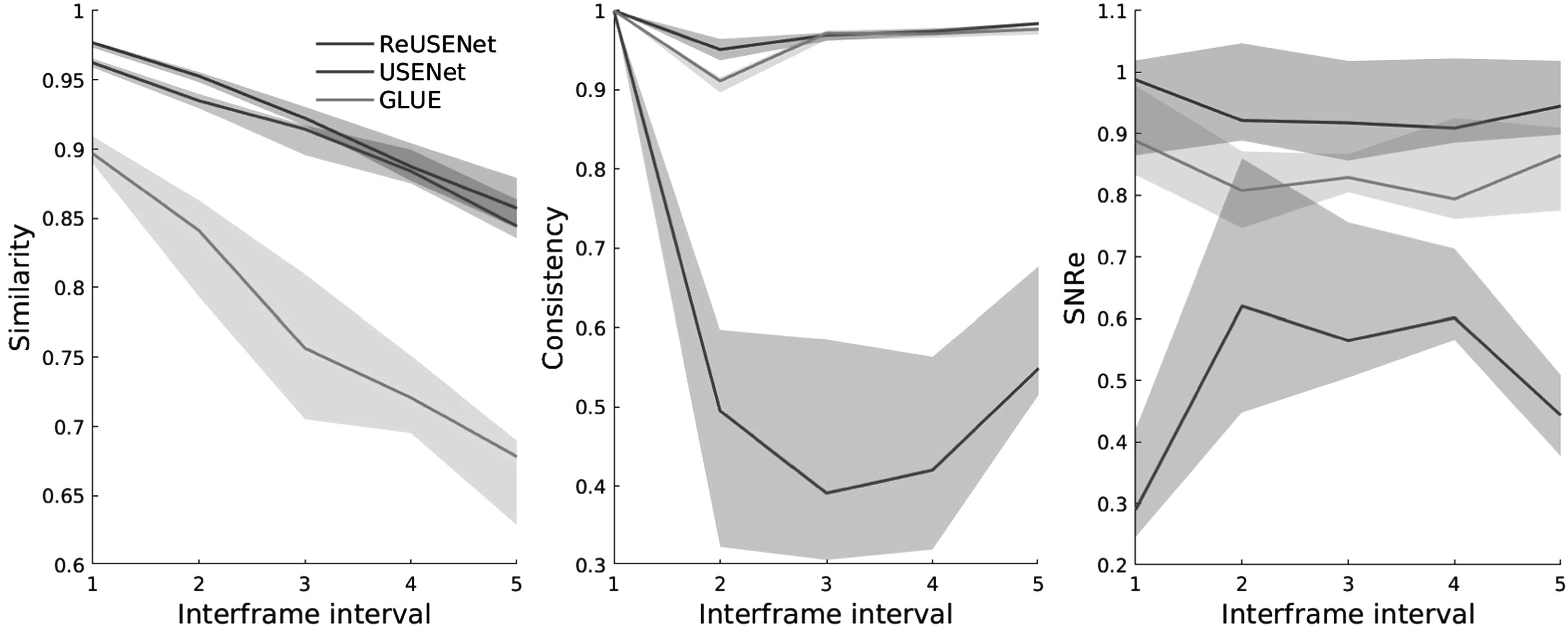
Similarity, consistency and SNRe scores with 25th percentiles for the *in vivo* testing dataset (*N* = 20) according to the interframe interval.

**Figure 8.**
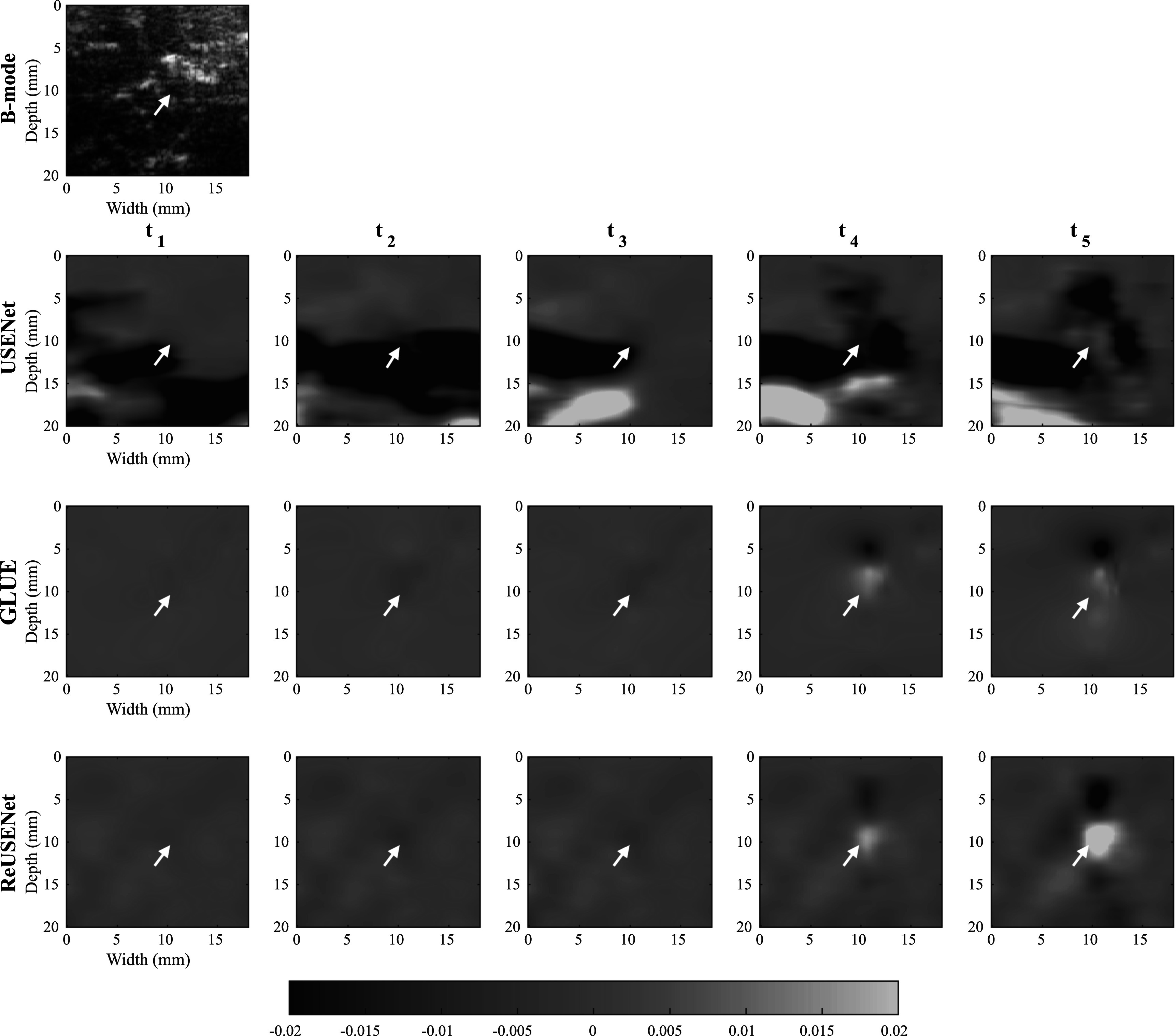
Temporal strain image sequence from the testing *in vivo* dataset.

Figures 6 shows an example of strain estimations of a temporal sequence computed by USENet, GLUE and ReUSENet. The white arrows indicate a blood vessel that is also visible in the B-mode image. The similarity, consistency and SNRe scores are represented in figure 7. Figure 8 displays an additional example of temporal strain estimation. Similar to the numerical phantom experiment, the consistency, similarity and SNRe scores for the USENet gradually decreases as the interframe interval increments. Average scores for ReUSENet (Similarity = 0.92 ± 0.03, Consistency = 0.96 ± 0.04, SNRe = 0.96 ± 0.2) are better than GLUE (Similarity = 0.77 ± 0.07, Consistency = 0.92 ± 0.10, SNRe = 0.88 ± 0.25). The mean TREs for individual cases, measured in pixels, are summarised in table 1. The average TRE and standard deviation for the entire testing dataset are the lowest for ReUSENet (2.87 ± 1.31), as compared with GLUE (3.80 ± 1.44) and USENet (4.37 ± 2.03).

**Table 1.** Registration accuracy of USENet, GLUE and ReUSENet. The mean TRE is calculated in pixels for all cases (8 landmark pairs per cases) from the *in vivo* testing dataset.

Case	Initial	USENet	GLUE	ReUSENet
1	6.98	5.02	4.05	3.29
2	6.48	4.08	3.12	2.57
3	10.00	4.21	3.05	1.05
4	8.62	4.15	4.42	2.48
5	3.22	4.82	2.91	3.44
6	7.65	3.09	3.07	2.18
7	3.92	3.50	3.33	4.03
8	9.91	5.05	4.91	2.61
9	9.17	4.62	3.64	3.92
10	7.50	3.39	3.24	2.80
11	8.80	3.89	2.83	1.77
12	9.90	4.88	3.61	3.55
13	8.18	2.58	2.32	2.35
14	6.83	4.64	4.68	3.62
15	8.00	2.62	4.24	1.20
16	10.45	9.16	9.00	7.45
17	4.05	2.59	2.28	2.32
18	5.58	2.15	2.66	1.85
19	5.92	2.49	3.57	2.37
20	18.06	10.40	5.12	2.94
**Mean**	**7.96**	**4.37**	**3.80**	**2.87**
**Stddev**	**3.09**	**2.03**	**1.44**	**1.31**

### 3.3. Open-source real-time visualisation module

As part of the open-source implementation of our method, we also introduce a real-time visualisation module, named DeepUSE, by using 3D-slicer, an open-source software platform dedicated to medical image processing and visualisation (Fedorov *et al* 2012). 3D-slicer is cross-platform, e.g. available for Windows, Mac OSX and Linux operating systems, and is built on the well-known VTK and ITK libraries. In addition, the platform is built to facilitate customisation and is used by a large and active international community. 3D-slicer also provides a Python interpreter which allows the use of python libraries and open-source machine learning framework such as PyTorch and Tensorflow.

DeepUSE is written in Python using the *ScriptedLoadableModule* base class provided by 3D-slicer. The module is fully integrated with the PyTorch implementation of both ReUSENet and USENet. DeepUSE’s features include the loading of a trained model via a configuration file, the offline inference of a loaded RF data sequence and the real-time inference of a stream of RF data sent using the OpenIGTLink protocol (Tokuda *et al* 2009). In terms of visualisation, the strain image nodes are automatically displayed alongside the RF data converted into B-mode to facilitate data interpretation.

## 4. Discussion

In this paper, two different neural networks were presented—USENet and ReUSENet. The former is a feed-forward encoder–decoder, which takes a pair of images as input, while the latter has a recurrent architecture with decoding convLSTM units that allows a temporal RF data sequence to be used as input. Both networks were trained in an unsupervised way, which allows fine-tuning on *in vivo* data. The two networks were compared with a supervised network (RFMPWCNet) and a state-of-the-art optimisation-based method (GLUE).

Our results suggest that incorporating temporal continuity by using convLSTM units improves displacement accuracy, especially for larger deformations. Experiments on numerical phantoms have highlighted the poor performances of standard feed-forward networks, such as USENet and RFMPWCNet, to estimate large range deformations. Indeed, they failed to estimate accurate displacement fields for strain level higher than 1.5% for RFPWCNet and 3.5% for USENet. On the other hand, ReUSENet utilises previous predictions to accurately estimate larger deformation (up to 4.5%). To the best of our knowledge, this is the first learning-based method to quantitatively reach the reported performance on such a large displacement search range.

Our results from the *in vivo* dataset showed that ReUSENet exhibited higher scores than USENet and GLUE in terms of SNRe, similarity and consistency. Most interestingly, the performance gap increased with the interframe interval, which may suggest that the recurrent network did make use of previous memory state to predict the current displacement. The TRE results also suggest that ReUSENet performs better than GLUE and USENet in terms of registration accuracy. Most interestingly, the performance gap increases with the interframe interval, which suggest that the sequential information enabled by the recurrent network improved the displacement estimation. It is important to note that the results from GLUE, RFPWCNet and USENet could have also been improved by applying the intermediate displacement fields at each time steps. There is however no published best practice on how to exploit temporal context with these method. We thus considered such possible extensions as out of scope for this work and only compared to published baselines.

We found that GLUE was sensitive to its regularisation parameter, *α* and *β*, which respectively control the displacement field smoothness in axial and lateral directions. We used the default parameters suggested by the authors for the simulation dataset, i.e. *α* = 5 and *β* = 1 (Hashemi and Rivaz 2017). However, those parameters tended to over-smooth the strain field for the *in vivo* dataset, which improved significantly the SNRe but also concealed the blood vessels in our experiments. Finding the optimum parameters for each cases may be possible, but can be too time-consuming for real-time applications. Therefore, we selected the same parameters (*α* = 2 and *β* = 0.1) for the entire testing dataset by visually inspecting the collection of output rather than automatically selecting the parameters that gave the better metric scores. Automating this process for optimisation-based elastography methods would be an interesting future research direction.

In conventional scanners, the strain elastogram is usually displayed next to, or directly overlayed, onto the B-mode images. Therefore, processing time and real-time visualisation is of high importance in quasi-static elastography. Both ReUSENet and USENet were able to achieve an inference speed of up to 20 frame-per-second (fps) on the DeepUSE Slicer extension, with a 12 GB NVIDIA GTX-1080ti GPU. For comparison, RFMPWCNet achieved a frame-rate of 6 fps on the same GPU. The inference speed difference between ReUSENet and RFMPWC-Net can be partly explained by the input size, i.e. RFMPWCNet takes as input 3-channel images, but also the number of parameters. ReUSENet consists of 1.5 millions parameters (0.8 millions for USENet), whereas RFMPWCNet has approximately 9 million parameters. The Matlab implementation of GLUE computed the strain field between one image pair in about 2 s on an Intel Core i7-7700HQ CPU. A GPU implementation of GLUE would significantly decreased the reported computation time.

Finally, we have shown that the ability to incorporate temporal information in a neural network for quasi-static elastography can increase the robustness to decorrelation noise and improve displacement estimation between pair of images that are temporally distant. We have also shown that including intermediate frames allows the recurrent network to measure larger deformation. Quasi-static elastography is highly user-dependent, and the displacement between each frame can not only be significant, but also variable, especially when the images are acquired at a high-frame-rate. The use of a recurrent network that encodes the spatio-temporal information coupled with a frame-selecting method could also improve real-time visualisation. Addressing temporal continuity in quasi-static elastography could also be of interest when using ultrafast ultrasound imaging technologies, i.e. plane wave imaging, to model fast tissue deformation (Porée *et al* 2015).

## 5. Conclusion

In this work, we present a new learning-based method for the estimation of strain elastograms between a pair of ultrasound RF data undergoing an axial compression. The proposed training scheme is unsupervised and we showed that it can be used to train a network directly on our open-access *in vivo* dataset of RF data of a human forearm. We also demonstrated that the use of recurrent units improves displacement estimation and temporal continuity for strain field predictions. The open-source code and 3D-slicer visualisation module are both publicly available. The inference speed of both networks can reach 20 frames per second on a 12 GB NVIDIA GTX-1080ti GPU. Therefore, it is highly suitable for real-time imaging and represents a great potential for the use of learning-based methods in quasi-static elastography.
